# Sleep endophenotypes of schizophrenia: slow waves and sleep spindles in unaffected first-degree relatives

**DOI:** 10.1038/s41537-018-0045-9

**Published:** 2018-02-09

**Authors:** Armando D’Agostino, Anna Castelnovo, Simone Cavallotti, Cecilia Casetta, Matteo Marcatili, Orsola Gambini, Mariapaola Canevini, Giulio Tononi, Brady Riedner, Fabio Ferrarelli, Simone Sarasso

**Affiliations:** 1 Department of Health Sciences, University of Milan, Milan, Italy; 2 Department of Mental Health, San Paolo Hospital, Milan, Italy; 3 Department of Psychiatry, University of Wisconsin, Madison, USA; 4 Department of Psychiatry, University of Pittsburgh, Pittsburgh, USA; 5 ‘L. Sacco’ Department of Biomedical and Clinical Sciences, University of Milan, Milan, Italy

## Abstract

Sleep spindles and slow waves are the main brain oscillations occurring in non-REM sleep. Several lines of evidence suggest that spindles are initiated within the thalamus, whereas slow waves are generated and modulated in the cortex. A decrease in sleep spindle activity has been described in Schizophrenia (SCZ), including chronic, early course, and early onset patients. In contrast, slow waves have been inconsistently found to be reduced in SCZ, possibly due to confounds like duration of illness and antipsychotic medication exposure. Nontheless, the implication of sleep spindles and slow waves in the neurobiology of SCZ and related disorders, including their heritability, remains largely unknown. Unaffected first-degree relatives (FDRs) share a similar genetic background and several neurophysiological and cognitive deficits with SCZ patients, and allow testing whether some of these measures are candidate endophenotypes. In this study, we performed sleep high-density EEG recordings to characterise the spatiotemporal features of sleep spindles and slow waves in FDRs of SCZ probands and healthy subjects (HS) with no family history of SCZ. We found a significant reduction of integrated spindle activity (ISAs) in FDRs relative to HS, whereas spindle density and spindle duration were not different between groups. FDRs also had decreased slow wave amplitude and slopes. Altogether, our results suggest that ISAs deficits might represent a candidate endophenotype for SCZ. Furthermore, given the slow wave deficits observed in FDRs, we propose that disrupted cortical synchronisation increases the risk for SCZ, but thalamic dysfunction is necessary for the disorder to fully develop.

## Diagnostics: Can sleep forewarn schizophrenia susceptibility?

Sleep EEG recordings of schizophrenia patients’ relatives differ from healthy norms, suggesting a new hereditary biomarker of disease. Leading a collaboration of Italian and US scientists, the University of Milan’s Armando D’Agostino, Anna Castelnovo and Simone Sarasso compared the electrical activity of healthy subjects’ brains during sleep to that of first-degree relatives of schizophrenia patients. The team found a significant reduction in a parameter related to the amplitude of “sleep spindles,” one of the two major types of brainwaves in non-REM sleep, in the relatives’ brains. However, the density of sleep spindles—a schizophrenia-associated trait—was unaffected in relatives. The authors suggest that reduced sleep spindle amplitude may help to indicate an individual’s risk of developing schizophrenia. Other EEG features from relatives suggest a communication impairment between neuron groups, which may also predispose the illness.

## Introduction

During sleep, the brain structures responsible for the gating of sensory information (thalamus) and the processing and response to input (cerebral cortex) are active and reciprocally communicating to produce the major spontaneous EEG oscillations of sleep: slow waves and spindles. These oscillations are a sensitive indication of brain physiology and their recording does not require effort, attention or concentration from the subject.

Sleep spindles, a defining characteristic of stage 2 non-Rapid Eye Movement sleep (N2), are brief powerful bursts of synchronous 12–15 Hz neuronal firing in thalamo-cortical networks.^1^ On the other hand, slow waves are the most prominent EEG feature of sleep and are defined as slow potentials with a frequency of 1–4 Hz and a relatively high amplitude (>75 μV), which represent periods of synchronous firing and silence across large populations of cortical neurons.^2^

Reductions of sleep spindles have been repeatedly and consistently observed in subjects with chronic, medicated Schizophrenia (SCZ)^,^^3–7^ and in nine adolescents diagnosed with an early onset SCZ.^8^ This deficit may reflect an abnormality in the function of a specific brain structure, the thalamic reticular nucleus (TRN), that is also involved in regulating attention and in the processing of sensory information during wakefulness.^9,10^ Some studies with small samples reported increased^11^ or unmodified^12–15^ spindle counts in drug-naive subjects. However, impaired spindling has been reported in unmedicated, early-course SCZ patients relative to early-course patients with other psychotic disorders and healthy control subjects.^16^

A number of studies also reported a decrease in delta power,^17,18^ in the number of slow-wave events^19^ or in slow-wave amplitude^20^ during sleep in patients with SCZ. However, these oscillations received less attention, possibly due to the partial inconsistency of reports, characterised by contradictory findings^4^ or aspecific trends.^16,19^ One possible explanation might be related to the counfounding effect of antisychotic drugs, that are well-known to induce EEG slowing^21^ and influence slow-wave activity (SWA, i.e., the EEG power in the slow-wave range) during sleep.^22^ Indeed, the largest study on unmedicated SCZ showed a reduction in slow waves,^17^ while the largest study on medicated SCZ patients^4^ showed no differences between groups. According to a recent meta-analysis, the slow-wave deficit manifests itself with the illness progression and is associated with negative and cognitive symptoms.^23^

As such, first-degree relatives (FDRs) of subjects diagnosed with SCZ represent a population of exceptional interest for several reasons, among which (1) the shared genetic background with affected relatives; (2) the partially overlapping neurophysiological, structural, neurofunctional and neurocognitive abnormalities^24^; (3) the absence of medication-related biases. Furthermore, microstructural sleep data obtained with new generation, dense-array EEG technology in this population are lacking.

We therefore conducted a high-density sleep EEG study to examine the oscillatory activity of the sleeping brain in healthy FDRs of SCZ patients compared to healthy subjects with no family history of SCZ.

## Results

### Clinical and demographic characteristics

A sample of 16 SCZ FDRs was evenly distributed in terms of gender (eight males and eight females) and had a mean age of 48.5 ± 14.2 and a mean education of 14 ± 3. None of the FDRs had relevant medical comorbidities and only two were on continuous treatment with cardiologic drugs (one with Sodium Fosinopril, Hydrochlorothiazide and Indobufene, the other with Amlodipine). Eleven subjects were siblings and five were parents of patients diagnosed with SCZ. Mean scores for Epworth Sleepiness Scale (ESS, 6.87 ± 2.45) and Pittsburgh Sleep Quality Index (PSQI, 3 ± 2.76) were within normal ranges, confirming the absence of subjectively assessed sleep problems. The control sample of 16 subjects with no personal or family history for psychiatric disorders was matched in terms of age (49.8 ± 12.7) and gender (8M–8F).

### Sleep architecture

Sleep architecture differences between the two groups are shown in Table 1. Percentage of REM, N1 and N3 sleep differed significantly between groups. FDRs showed average 6% reductions of both REM and N3 sleep in favour of a >10% increase of N1. Sleep efficiency was reduced by 9% in FDRs, whereas total sleep time (TST) presented a 25% reduction in FDRs compared to the healthy control group. Sleep latency, REM latency, wake after sleep onset (WASO) and percentage of N2 did not differ between the groups. Given the differences in sleep architecture between the groups, separate analyses were performed on the first NREM cycle, which showed a comparable duration between FDRs and healthy controls (131.18 ± 16.8 and 119.53 ± 9.3 min, respectively; *p* = 0.55) and showed no differences in N2/N3, the NREM sleep stages utilised for all EEG data analyses.

**Table 1:** Sleep macrostructural differences between the two groups

Sleep parameters	Mean FDRs (±SE)	Mean controls (±SE)	*t*-test (*p*)
TST (min)	274.3 ± 19.3	368.1 ± 9.04	<0.05
WASO (min)	84.63 ± 10.4	67.22 ± 8.1	n.s.
Sleep efficiency	75.80 ± 2.5	84.56 ± 1.8	<0.05
N1 (%)	14.20 ± 2.7	3.53 ± 0.6	<0.05
N2 (%)	51.75 ± 2.3	49.37 ± 1.9	n.s.
N3 (%)	19.49 ± 1.4	25.89 ± 2.1	<0.05
REM (%)	14.55 ± 1.7	21.21 ± 0.9	<0.05
REML (min)	119.57 ± 14.8	96.38 ± 9.1	n.s.
Cyc1 (min)	131.18 ± 16.8	119.53 ± 9.3	n.s.
N1 cyc1 (%)	5.59 ± 1.4	1.19 ± 0.2	<0.05
N2 cyc1 (%)	18.35 ± 4.1	11.20 ± 1.3	n.s.
N3 cyc1 (%)	8.03 ± 1.2	11.84 ± 1.9	n.s.

### EEG power, spindle and slow-wave analysis

**Fig. 1:**
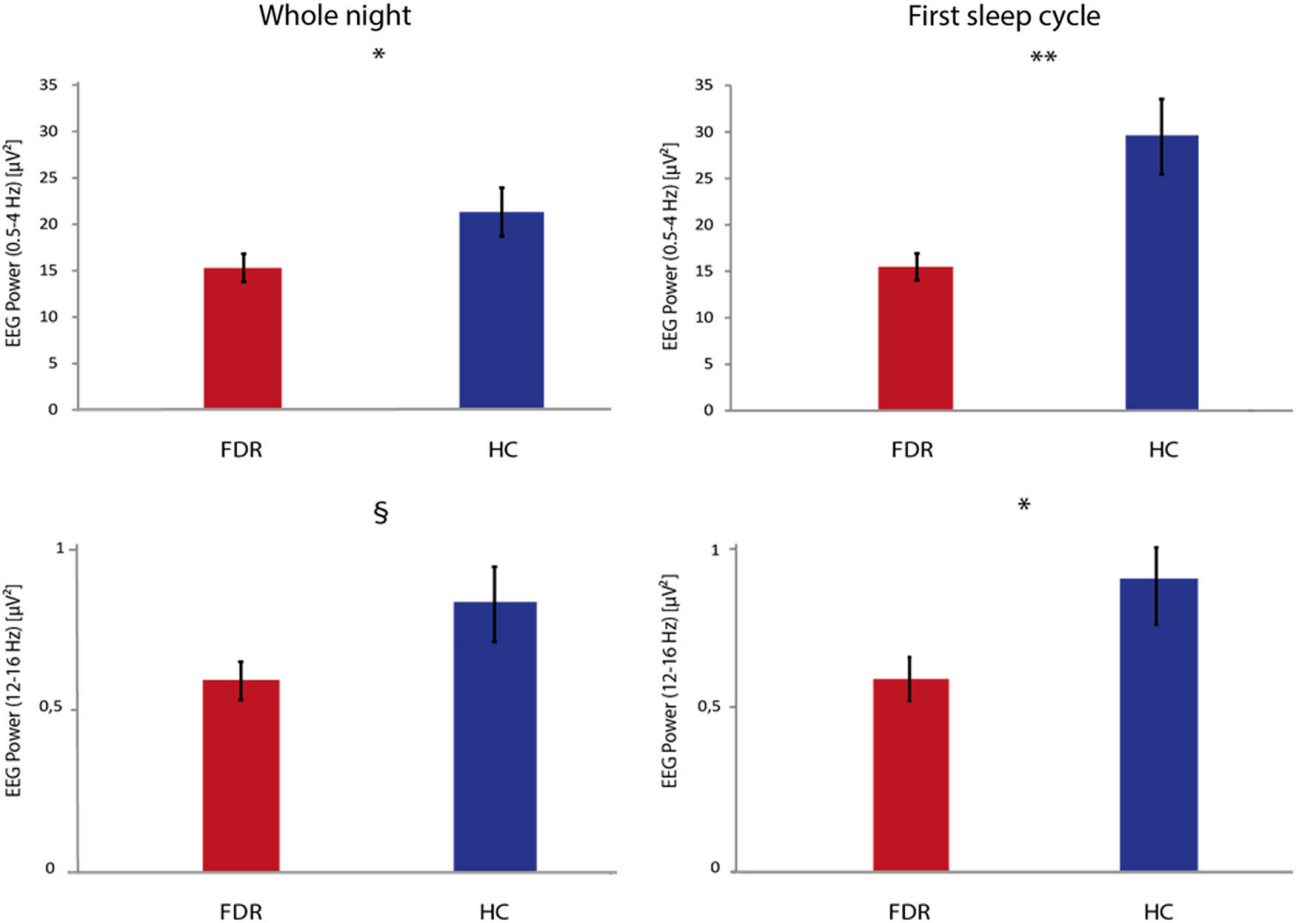
EEG spectral power in first-degree relatives (FDR) of Schizophrenia patients and healthy control (HC) subjects. Top row: mean group spectral power differences in the delta frequency range over the whole night (left) and during the first cycle (right). Bottom row: mean group spectral power differences in the spindle frequency range over the whole night (left) and during the first cycle (right). *p < 0.05, **p < 0.01, ^§^*p* < 0.1

EEG power was calculated for the two groups over the whole night and during the first cycle of sleep. Significant group differences were observed for both slow waves and sleep spindles frequency ranges, particularly during the first cycle (Fig. 1).

**Fig. 2:**
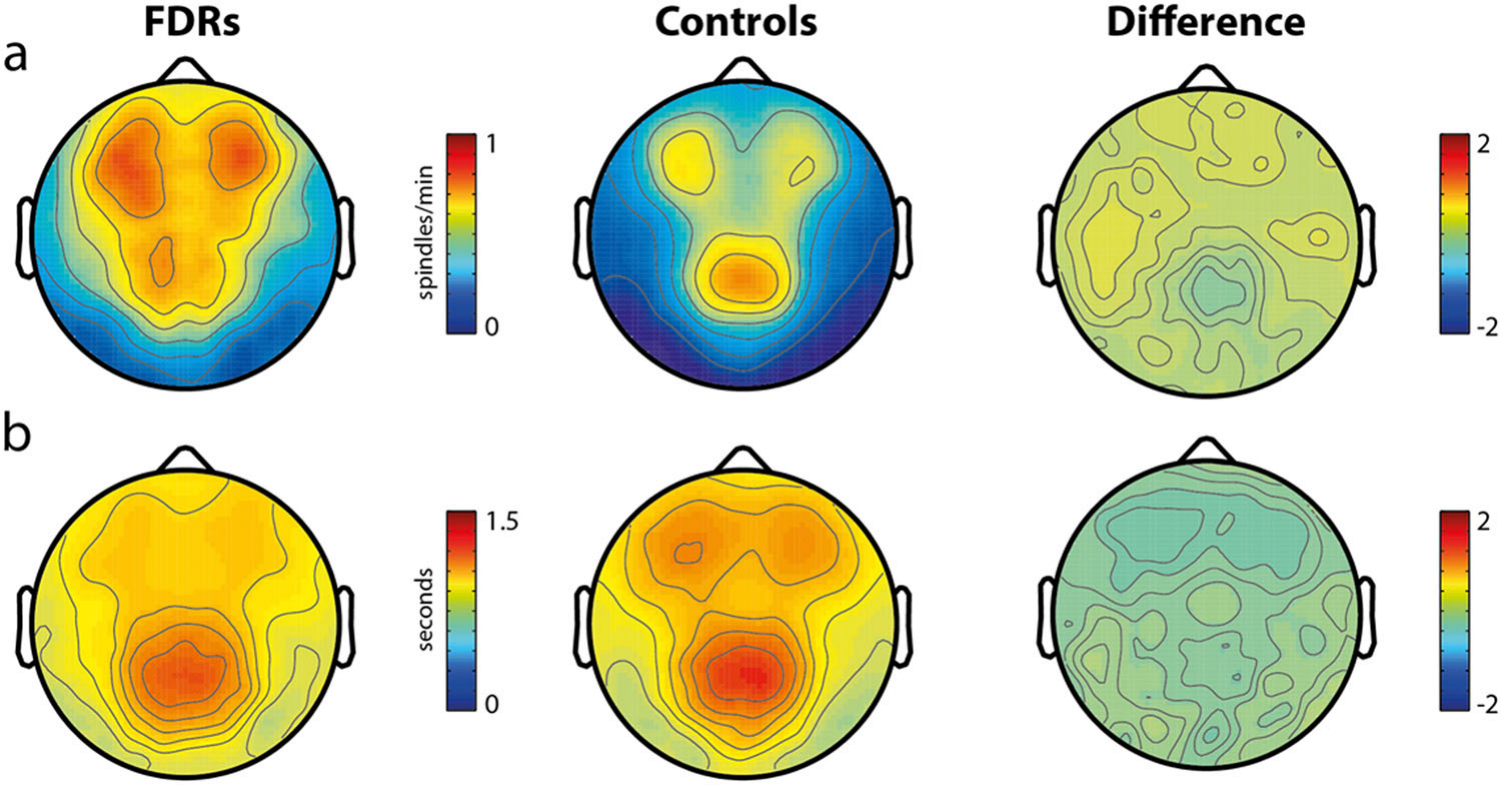
**a** Whole-night sleep spindle density in first-degree relatives (FDR) of Schizophrenia patients and healthy control (HC) subjects. Topographical distribution of spindle activity in both groups confirms validity of the methodology employed. No statistically significant difference could be observed between FDR and HC groups in the whole sigma frequency range. Further analyses (topographies not shown) failed to detect significant between-group differences also for fast and slow spindle frequency ranges. **b** Whole-night sleep spindle duration in first-degree relatives (FDR) of Schizophrenia patients and healthy control (HC) subjects. No statistically significant difference could be observed between FDR and HC groups in the whole sigma frequency range. Further analyses (topographies not shown) failed to detect significant between-group differences also for fast and slow spindle frequency ranges

**Fig. 3:**
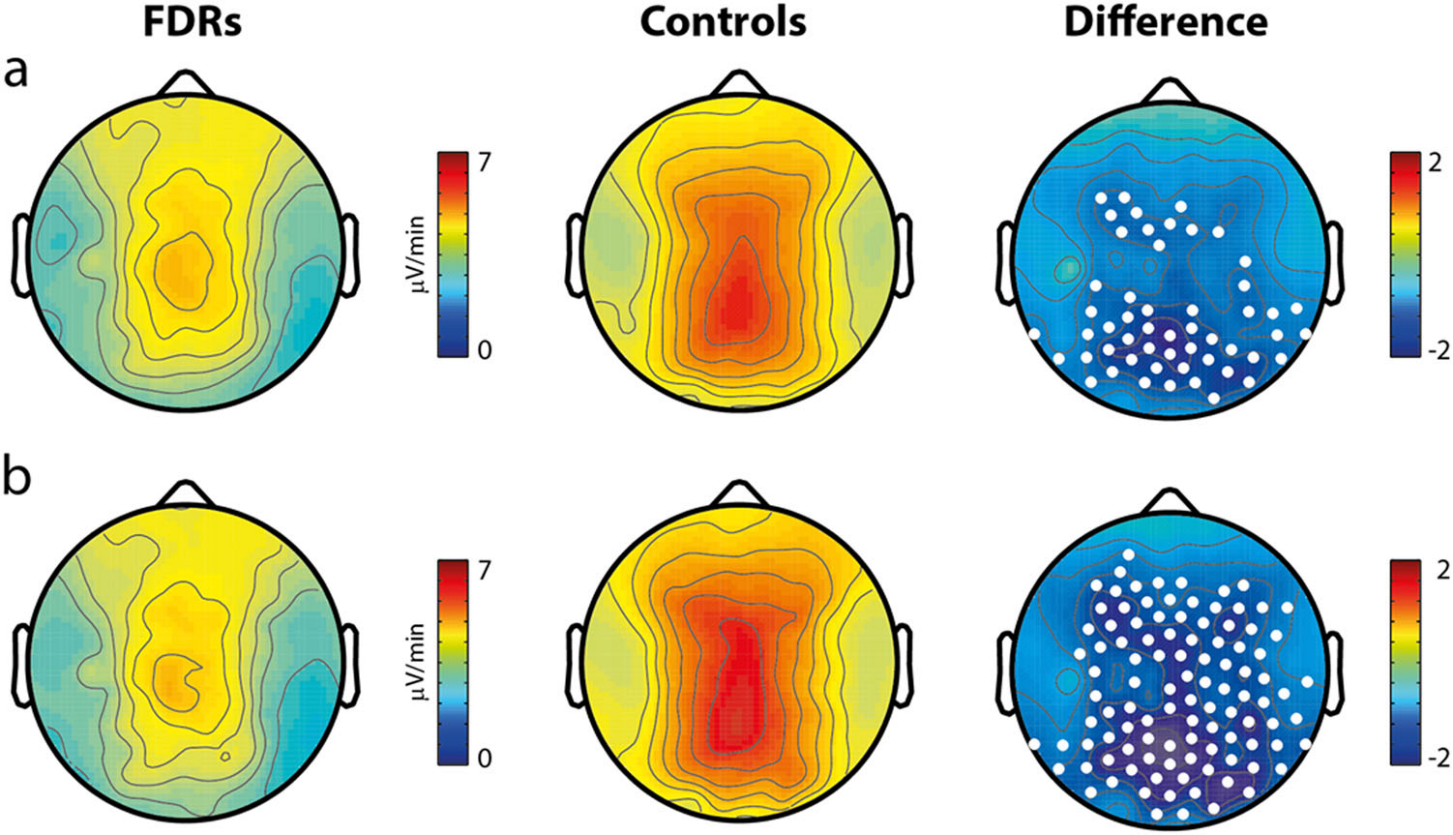
Integrated spindle activity deficit in first-degree relatives (FDR) of Schizophrenia patients and healthy control (HC) subjects. Significant differences were observed between FDR and HC groups in the whole sigma frequency range for ISAs. The strength of this finding was confirmed over the whole night (**a**), and during the first cycle (**b**). White dots on right-sided topographies indicate electrodes showing significant differences between the two groups

An initial analysis was performed for spindle density, duration and integrated spindle activity (ISAs) in the 12–16 Hz frequency range for the whole night. The topography of each parameter was similar between groups, with peaks in prefrontal and centroparietal areas. Spindle density (Fig. 2a) and duration (Fig. 2b) in these regions did not differ between groups. A significant deficit of ISAs compared to healthy subjects was present in FDRs at a cluster level (*p* < 0.05) in centroparietal regions (Fig. 3, top row). Additional analyses were performed for slow (12–14 Hz) and fast (14–16 Hz) spindles. ISA was significantly reduced in centroparietal regions for both frequency ranges.

The same analysis was then restricted to the first cycle. Again, topographical distribution of spindles peaked in prefrontal and centroparietal regions in both groups. Spindle density and duration overlapped between groups, yielding no significant difference. The significant ISAs deficit observed in the FDR group in the whole-night analysis was also confirmed for a larger cluster located in centroparietal regions (*p* < 0.05) during the first cycle (Fig. 3, bottom row).

All spindle parameters were then re-analysed restricted to whole-night N2. Density and duration findings on whole-night and first cycle were confirmed. Likewise, reduced ISAs was confirmed in centroparietal regions for the FDR group (result not shown).

Additional analyses were performed for slow (12–14 Hz) and fast (14–16 Hz) spindles. ISA was significantly reduced in centroparietal regions for both frequency ranges.

**Fig. 4:**
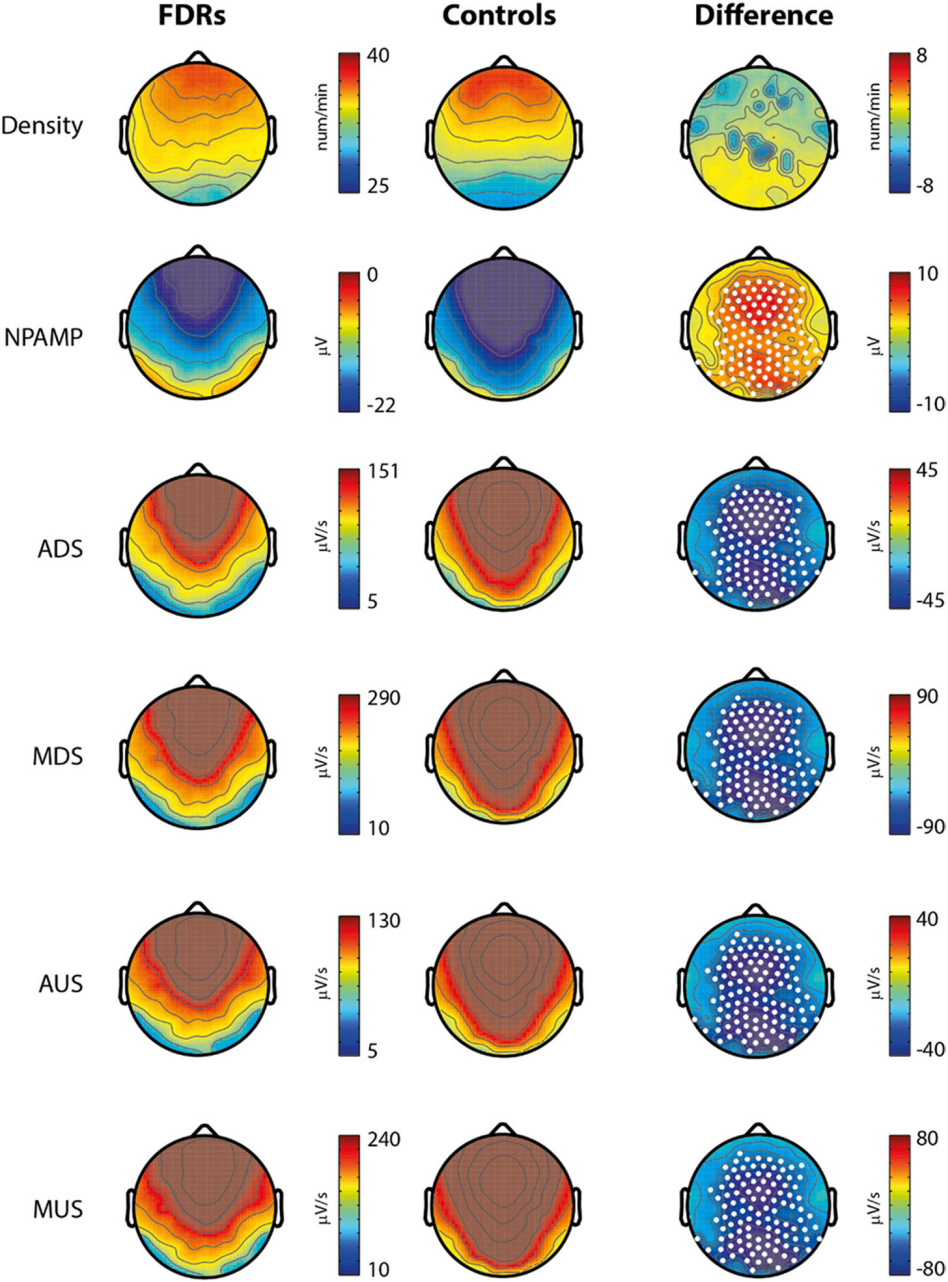
Slow Wave (SW) parameters analysis—all night Left: Plots of each averaged parameter for FDRs in all-night setting. Centre: Plots of each averaged all-night parameter for control subjects in all-night setting. Right: Mean group differences. Statistically significant channels are marked as white dots. NPAMP negative peak amplitude, ADS average down-slope, MDS maximal down-slope, AUS average up-slope, MUS maximal up-slope

Slow-wave density did not differ significantly between the two groups both during the whole-night and within the first cycle (Fig. 4 and Supplementary Figure 1). All other measured parameters, i.e., negative peak amplitude (NPAMP), average down-slope (ADS), maximal down-slope (MDS), average up-slope (AUS) and maximal up-slope (MUS), showed marked, significant and diffuse deficits in FDRs compared to healthy controls (*p* < 0.05) both during the whole-night and within the first cycle.

When slow-wave parameters were re-analysed restricted to whole-night N2 and N3, density did not differ but mean amplitude and slope parameters remained significantly impaired in the FDR group (result not shown).

### Measures of neurocognition and psychosis proneness

No significant correlation was observed between available neuropsychological tests and ISAs values nor with slow-wave parameters. Spindle deficit also failed to yield any significant correlation with psychosis proneness scores in either the delusional thinking (2.62 ± 2.8) or hallucination (4.43 ± 5.6) scores. On both scales, all participants scored below the lower thresholds for psychosis. Mean BACS equivalent scores were within normality ranges for verbal memory (3.37 ± 0.80), digit sequencing (2.69 ± 1.19), symbol coding (2.44 ± 1.55), verbal fluency (2.19 ± 1.60) and Tower of London (2.44 ± 1.55) subtests and borderline pathological for the Token motor test (1.44 ± 1.67). No significant correlation with any BACS subtest was observed for any of the identified clusters. No correlation was found between BACS and psychosis proneness scores.

## Discussion

In this study, we employed dense-array EEG technology to investigate whole-night sleep in adult FDRs of SCZ patients with no psychiatric diagnosis or treatment.

### ISAs is a sleep endophenotype for SCZ

The main finding of this study is that FDRs of subjects diagnosed with SCZ have a reduction in ISAs, which reflects spindle amplitude. This reduction reached statistical significance over centroparietal regions for both slow and fast spindles during N2/N3. This is the first study to evaluate such a marker of thalamocortical function in a population of healthy, adult relatives of SCZ patients. This finding suggests that ISAs might be employed as a sensible parameter to indicate a predisposition to develop SCZ. One previous study reported a significant reduction of sleep spindle amplitude in 17 children and two siblings of SCZ patients.^16^ However, almost 70% of these children (13 out of 19, mean age 14 ± 4) had a lifetime history of other major mental disorders as attention-deficit/hyperactivity disorder (*n* = 5), major depression (2), separation anxiety disorder (2), oppositional defiant disorder (2) and conduct disorder (2), and at the time of the study one child was taking amphetamine/dextroamphetamine and another was taking sertraline. Furthermore, morphofunctional brain abnormalities observed in children and adolescent FDRs of psychotic patients cannot be unambiguously interpreted. Indeed, severe abnormalities of cortical thickness in siblings of very early-onset SCZ patients observed during childhood have been found to normalise by the age of 20 (see ref. ^25^).

### Spindle density is specific for the disease

The second major finding of the present work is that sleep spindle density is not reduced in SCZ FDRs. This finding indicates that this parameter is specific for the disease and confirms an essential role of the TRN, the spindle generator, in full-blown cases of SCZ. Indeed, the mean age of our FDR sample was clearly above the superior threshold for the disorder to develop. Our finding is partially in line with Manoach et al.,^16^ who found a trend towards a reduction of sleep spindle density that didn’t reach statistical significance. The influence of several major psychiatric disorders in their FDR sample might account for the observed trend. On the other hand, another study recently found a deficit restricted to fast spindle density in 13 adult, unaffected FDRs.^26^ In this sample, neither the duration nor the amplitude was significantly impaired, although both parameters were inbetween the SCZ and the healthy control group. The authors employed an individual adjustment method, with spindles detected at the exact frequency bands of the given individual and as low as 9 Hz,^27^ rather than at the fixed 12–16 Hz frequency algorithm used by our group. Although some slower spindling activity might be lost, this latter threshold appears to reflect the consensus definition of a sleep spindle—a train of distinct waves with frequency 11–16 Hz (most commonly 12–14 Hz^28^)—more accurately. In summary, spindle amplitude and ISAs in particular should be considered a valuable endophenotype for SCZ, although current sample size limitations and differences in spindle detection algorithms employed across groups warrant further larger investigations to confirm this finding.

### Sleep spindle and slow-wave deficit in amplitude point to a general deficit in cortical connectivity and might predispose to the disease

Our study investigated SWA, that had never previously been explored in FDRs of SCZ. Significant differences emerged in slow-wave amplitude (nPAMP) and slope parameters (ADS, MDS, AUS, MUS). This finding points towards a cortical synchronisation impairment rather than towards a wave generation deficit. The slope of evoked waves is traditionally used as an electrophysiological measure of cortical synchronisation and synaptic strength.^29–31^ Here, the deficit was widespread whereas it was more specifically localised on centroposterior cortical regions on first-cycle N2N3 analyses. Given the preserved physiology of spindle and slow-waves density, the amplitude deficit observed in FDRs could reflect a partial cortical dysfunction that does not impact on spindle and slow wave generation but rather only on their morphology. On the other hand, smaller and shallower waves could reflect a more general problem of cortical connectivity, leading to an incomplete synchronisation of cortical neurons which determines a discharge over relatively limited neuronal populations.

### Limitations

Among the limitations of our study, the difference observed in sleep architecture between the two experimental samples is perhaps most relevant. However, this can be considered a characteristic of the target population, which is known to have a poorer sleep quality compared to the general population. Indeed, one previous study^32^ reported altered TST, stage shifts, stage 1 percentage of TST, stage 2 duration, stage 3 latency, stage 4 duration and percentage of TST, in line with our results. Similarly, Manoach et al.^16^ found a significantly worse sleep quality in FDRs. This was clearly observed as increased WASO and reduced sleep efficiency, a disruption in sleep architecture with a greater percentage of time in lighter sleep and trends towards lower percentages of slow wave and REM sleep. Crucially, slow wave and spindle densities were found to be normal in our sample, suggesting that differences in sleep architecture were unlikely to affect both spindle and slow-wave analysis results and that the electrophysiological deficit appears more 'qualitative' (i.e., in the graphoelement features) than 'quantitative'.

Another limitation of our study is the lack of comparable data on the cognitive profile of the two samples. Indeed, the normative sample of healthy subjects was drawn from two previous studies conducted with a different set of psychometric and cognitive assessment tools. Because this sample lacks some demographic and cognitive measures, no information is available on whether they were well-matched to FDRs on important features such as socioeconomic status or IQ. This might be considered a potential confound since IQ measures have been found to correlate with sleep spindles.^33^

The small sample size is another limitation of the reported study, albeit in line with the few previous publications including whole-night sleep data in FDRs of SCZ patients [19 (ref.^16^); 13 (ref. ^26^); 14 ref. ^32^)]. The limited number of subjects might also explain the lack of significant impairment observed in most BACS subtests and the absence of correlations with sleep parameters. Whereas motor task results were mildly pathological, all tests of memory, verbal fluency and executive function were within median values. Significant impairment of BACS scores had previously been reported in a large sample of SCZ relatives without a history of psychosis.^34^ Positive correlations have also recently reported between fast spindle density and early and late recall, and between slow spindle density and late recall measured by the Verbal Learning and Memory Test in 11 healthy FDRs.^26^ Indeed, sleep spindles have been associated with neurocognitive performance in several SCZ studies.^7,8^ Furthermore, decreased delta sleep has been associated with impairments in visuospatial memory,^35^ attention/cognitive flexibility^18^ and consolidation of declarative memory in SCZ.^30^ The cognitive measures employed here may not be sensitive enough to detect more subtle deficits and to correlate with sleep neurophysiological parameters that have been more classically associated with task-related learning and plasticity.^31,36^ However, the rigorous exclusion of any subject with a psychiatric or neurological history might also have contributed to the normal cognitive profile observed in our FDR sample.

To clarify the discrepancy with the existing literature, future studies will need to explore the relationship between sleep endophenotypes and subtle neurocognitive deficits in larger samples of SCZ FDRs with shared cognitive batteries.

Overall, these concerns limit the interpretation of the reported differences in spindle and slow-wave parameters.

## Conclusions

We have previously observed that sleep spindling might be considered a reliable biomarker for SCZ because other clinical conditions associated with spindle deficits either emerge during early neurodevelopmental stages (intellectual disabilities, inborn errors of metabolism, autism), have a typically advanced age of onset (Alzheimer’s Dementia or Parkinson’s Disease), or characteristic abnormalities of sleep-related behaviour (REM or NREM parasomnias).^37^ On the other hand, slow-wave deficits may suggest a disruption of cortical synchronisation mechanisms in FDRs. Although this might increase the risk for SCZ, aberrant thalamic activity is necessary for the disorder to fully develop. This preliminary observation should be replicated in larger samples and could legitimate further structural and functional studies on this topic.

## Methods

### Experimental sample

FDRs were recruited primarily by referral from patients or from their treating physicians, within the Department of Mental Health of the San Paolo University Hospital in Milan, Italy. Diagnoses of SCZ were confirmed independently by at least two experienced clinicians (A.D.′A., O.G.) through unstructured clinical interview, reviewing of medical charts and clinical conferences. Diagnoses were based on DSM-5^38^ criteria for Schizophrenia and relatives of patients with any other Schizophrenia spectrum diagnosis, including Schizoaffective disorder, were excluded. Healthy control participants were drawn from two other studies conducted at the University of Wisconsin—Madison sleep laboratory, both with the same recording system and similar procedures as the FDR group.

Inclusion criteria for all subjects were the ability to provide written consent prior to admission, age between 18 and 65 years; good general health determined by the investigator on basis of medical history, physical and neurological exam. Exclusion criteria for all subjects were a personal history of any major clinical condition, including psychiatric, neurological or sleep disorders; diabetes requiring insulin treatment; a relevant heart disorder; a diagnosis of cancer within the previous 3 years; positive history of alcohol/drug use problems within the previous year; regular night or late evening shift work; travel with time zone shifts >3 h in the month prior to participation; positive screening questionnaires for sleep disorders. None of the participants had taken any medication with psychotropic effects within the previous year. Control subjects were also excluded if they had any FDR with a history of major psychiatric disorders.

After a complete description of the study, written informed consent was obtained. The study was approved by the San Paolo Hospital ethics committee and by the University of Wisconsin Health Sciences Institutional Review Board.

### Experimental Procedure

All participants were interviewed to obtain a complete psychiatric, neurologic and medical history and to rigorously exclude any psychiatric diagnosis based on DSM-5 criteria.^38^ All subjects underwent overnight in-laboratory high-density EEG (hd-EEG) recording sampled at 500 Hz and collected with vertex referencing (256 channels; Electrical Geodesics Inc., Eugene, OR). Subjects were asked to arrive at the lab ~2 h before their usual time of falling asleep. Whole-night hd-EEG was performed with 256-electrode nets designed to improve electrode contact with the scalp, thereby enabling long-duration recordings (EGI, Eugene, Oregon, United States). The subject was accomodated in a sleep suite and allowed to sleep within 1 h of the self-reported bedtime until morning. All subjects were allowed to sleep undisturbed until their normal wake-up time in the morning.

Moreover, FDRs completed the following tests: (i) The Brief Assessment of Cognition in Schizophrenia (BACS),^39^ which provides a brief, reliable and valid test of global neuropsychological function that has already been used in SCZ endophenotype research.^34^ Tests included in the BACS are list learning, digit sequencing, verbal fluency, token motor task, symbol-coding and Tower of London. Normative values have been established using the Equivalent Scores method to enable comparison with other neuropsychological tasks commonly used in the assessment of the Italian population.^40^ (ii) PSQI^41^ and ESS^42^ were employed to assess subjective sleep quality and the presence / absence of relevant sleep disorders. (iii) Peters et al. Delusions Inventory (PDI), a self-administered test that is commonly used to assess psychosis proneness in the general population. FDRs completed the 21-item Italian version.^43^ PDI items address unusual subjective experiences and beliefs in the general population with questions in dubitative form. The optimal cut-off point for psychotic subjects has been set at 8, and the test has been shown to reliably distinguish between subjects with psychosis and subjects with other forms of mental disorder such as anxiety spectrum disorders. (iv) Finally, participants completed a revised version of the Launay–Slade Hallucination Scale.^44^ The questionnaire is commonly used in research settings to assess the prevalence of hallucinatory experiences in healthy subjects. FDR results were compared with available normative values.

### Sleep staging

Sleep staging was performed according to standard criteria,^28^ using Alice^®^ Sleepware (Philips Respironics, Murrysville, PA) based on 30-s epochs for 6 EEG channels (F3/A2, F4/A1, C3/A2, C4/A1, O1/A2, O2/A1). Submental electromyogram (EMG) and electroculograms (EOGs) were selected from channels around the neck, jaw and eyes.^45^ FDRs were also evaluated on a full set of neuropsychological and psychopathology measures, which are reported in the Supplementary data. EEG analyses were conducted on NREM Stages 2 an 3 (N2/N3) of the whole night and of the first cycle using MATLAB R2009b (The MathWorks Inc., Natick, MA). All EEG signals were high-pass filtered at 0.1 Hz, down-sampled to 128 Hz, band-pass filtered (0.5–40 Hz), and re-referenced to the average of the scalp voltage for all 256 channels. Clear artefactual 30-s epochs were visually excluded from sleep staging during the scoring procedure.

### EEG signal analysis

After high-pass and band-pass filtering, a previously reported artefact removal algorithm was used to reject 30-s epochs, which exceeded thresholds based on the mean power for each channel in 0.8–4.48-Hz and 20–30-Hz bands.^4^ Power spectral density of NREM epochs was then computed with a 0.16-Hz bin resolution, fast-Fourier transform routine (Welch’s averaged modified periodogram with a Hamming window, averages of five 6-s epochs).

An automated algorithm was used to detect sleep spindles. NREM epochs were band-pass filtered between 11 and 16 Hz and the amplitude of the rectified filtered signals were used as time series for each channel. Because signal amplitude varied significantly across channels, thresholds relative to the mean amplitude of each channel were used. The lower threshold was set at two times the mean amplitude of the channel signal and the upper threshold was set at eight times the mean amplitude. Whenever an amplitude fluctuation exceeded the upper threshold, a spindle was detected. Points preceding or following (≥0.25 s) this maximum when amplitude dropped below the lower threshold were considered as beginning and end of a spindle.

The following parameters were then considered: spindle duration, spindle density and ISAs. Duration was defined as the distance in seconds between the points immediately preceding or following the intersection between the time series and the lower threshold. Spindle Density was defined as the number of spindles per minute of NREM sleep and ISAs as the integration of the amplitude value of each spindle (normalised by its duration) divided by NREM sleep duration. Compared to other available measures, ISAs synthesises the average spindle activity over time, allowing to combine spindle amplitude, number and duration in one single parameter.

Slow wave detection procedures are similar to those employed in previous work on period-amplitude analysis;^46–48^ the automated algorithm employed has been reported in preexistent hd-EEG studies on slow waves in healthy subjects.^29^ The following parameters were then considered: slow wave density, NPAMP, and average/maximal slopes. Density was defined as the number of detected slow waves per minute of NREM sleep. NPAMP was the most negative point in between two zero-crossings. Average slope was defined as the amplitude of the most negative peak divided by the time from the previous zero crossing (first-segment, down-slope) or the time until the next zero crossing (second-segment, up-slope). Maximal slopes were defined as the maximum of the signal derivative (after applying a 50-ms moving average filter) between the negative zero crossing and the most negative peak (first-segment, down-slope), as well as between the most negative peak and the positive-going zero crossing (second-segment, up-slope).

### Statistical analyses

To compare demographic characteristics, sleep architecture and EEG power between groups, unpaired *t*-tests were performed. Group differences in the topographical distribution of spindle and slow waves parameters were assessed with statistical non-parametric mapping (SnPM), with supra-threshold cluster tests (Nichols and Holmes, 2002) to correct for multiple comparisons, using a threshold *t*-value (*t* = 2042, corresponding to *α* = 0.05 for the given degrees of freedom) with fixed number of combinations (2^Nsubjects^).^41,49,50^ Finally, Spearman’s rank correlation analyses were performed between sleep parameters and available neurocognitive tests and measures of psychosis proneness. Correlations were run at a single channel level for exploratory analysis and also at cluster level for clusters identified by spindles and slow waves analyses.

### Data availability

All the material will be available upon request from the corresponding authors.

### Code availability

Codes employed for spindle and slow wave analyses are available upon request.

## Electronic supplementary material

Supplementary Figure 1.
