# Supplementary material for: Sleep endophenotypes of schizophrenia: slow waves and sleep spindles in unaffected first-degree relatives
Source: NPJ Schizophr. 2018 Feb 9;4:2. doi: 10.1038/s41537-018-0045-9 (PMC5807540; doi:10.1038/s41537-018-0045-9)
Supplement: Supplementary file 1 — Supplementary Figure 1. [file 41537_2018_45_MOESM1_ESM.docx]

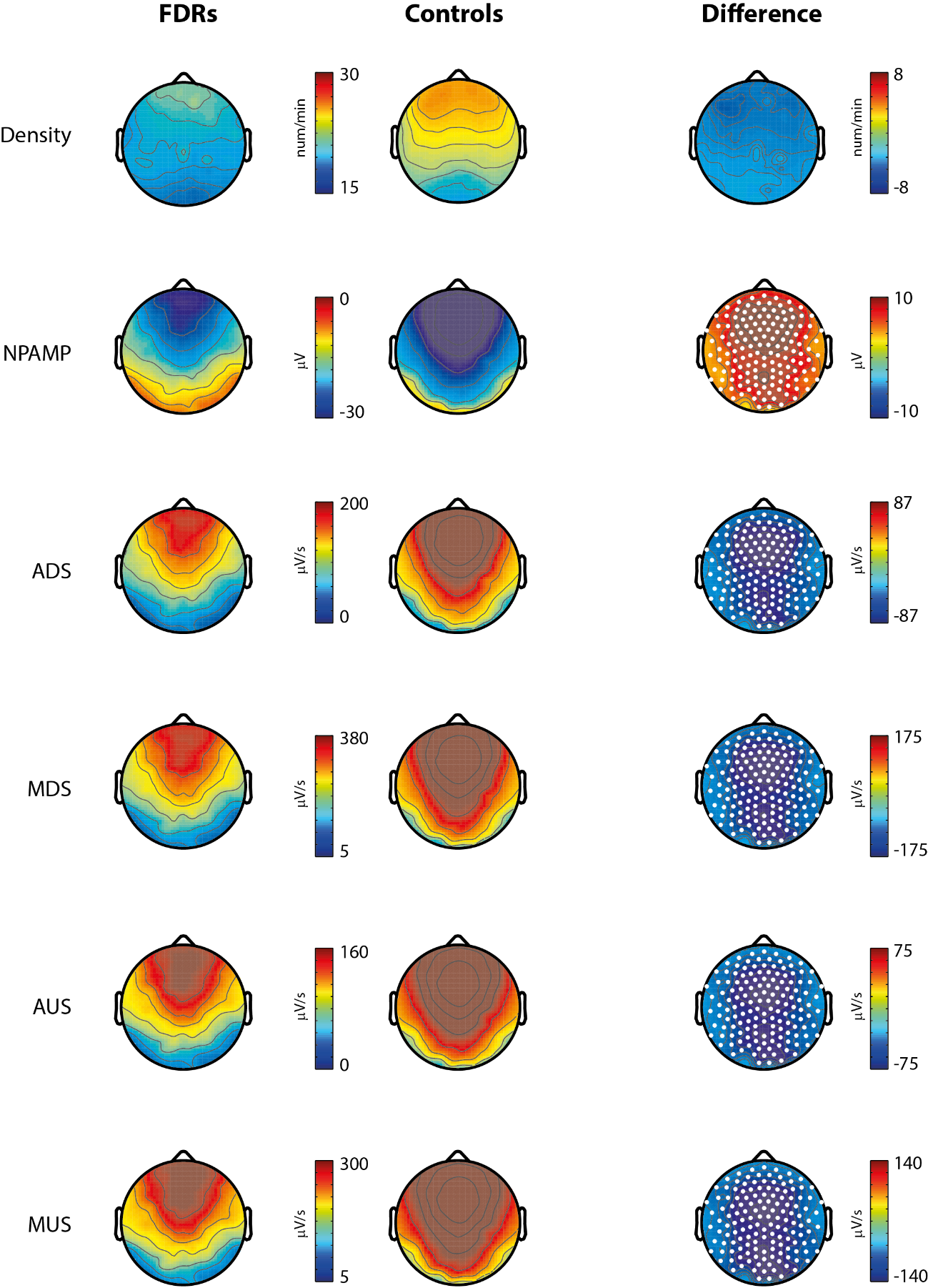

**Supplementary Figure 1.** Slow Wave (SW) parameters analysis – first cycle **Left:** Plots of each averaged parameter for FDRs in the first sleep cycle. **Center:** Plots of each averaged parameter for control subjects in the first sleep cycle **Right:** Mean group differences. Statistically significant channels are marked as white dots. NPAMP= negative peak amplitude; ADS= average down-slope; MDS= maximal down-slope; AUS= average down-slope; MUS= maximal up-slope.
